# Supplementary material for: A Cyanobacteria Enriched Layer of Shark Bay Stromatolites Reveals a New Acaryochloris Strain Living in Near Infrared Light
Source: Microorganisms. 2022 May 17;10(5):1035. doi: 10.3390/microorganisms10051035 (PMC9144716; doi:10.3390/microorganisms10051035)
Supplement: Supplementary file 1 [file microorganisms-10-01035-s001.zip › microorganisms-1669219-supplementary.pdf]

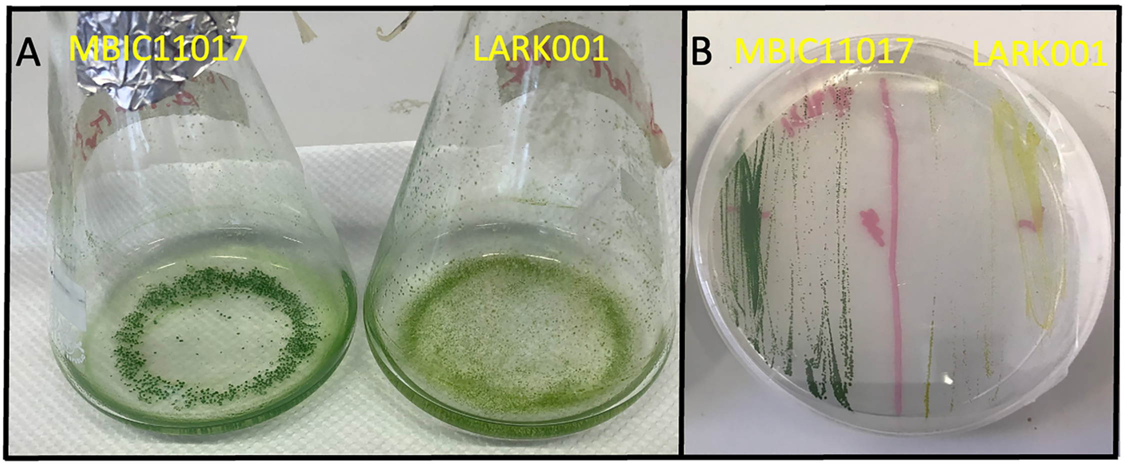

Figure S1. Visual appearance of the cultured strains. (A) Difference in colour and aggregation of each strain when grown in liquid cultures in NIR. (B) Difference in colour of the strains when grown on agar in NIR .

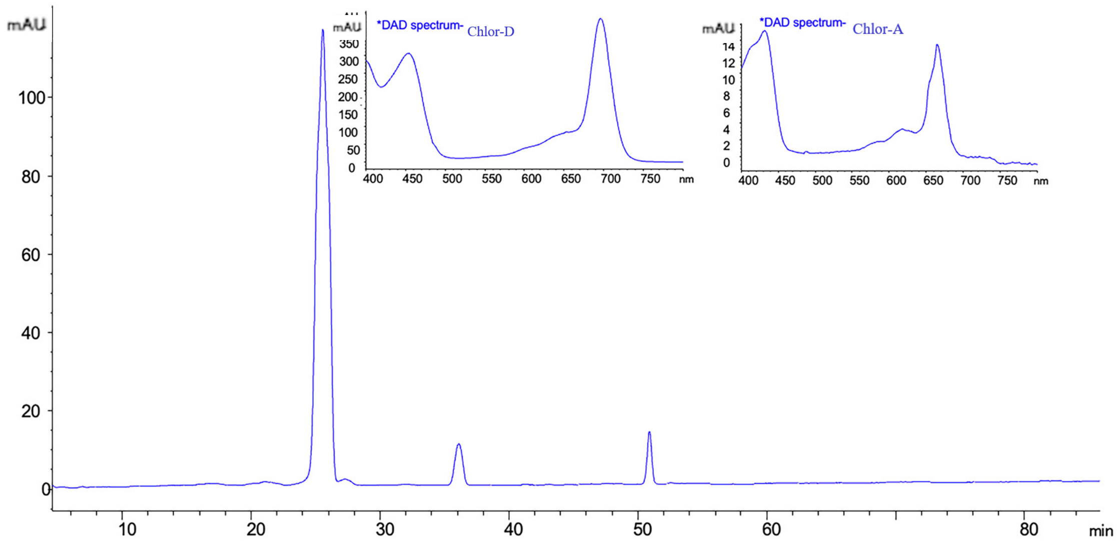

Figure S2. Pigment analysis of the *Acaryochloris* MBIC11017 and LARK0001 strains grown in liquid culture in white light and NIR. The top panel shows a representative HPLC chromatogram showing a major Chl *d* peak after 25 minutes retention and a minor Chl *a* peak after 36 minutes retention. Concentration of each peak for each strain at each light condition are summarized in the bottom table.

	Chl *a*	Chl *d*	Chl *a*/ Chl *d*	% Chl *d*
***Acaryochloris* MBIC11017 (WL)**	0.082409	0.857949	0.096054	91%
***Acaryochloris* MBIC11017 (NIR)**	0.032121	0.880435	0.036483	96.4%
***Acaryochloris* LARK001 (WL)**	0.052183	0.748497	0.069717	93.4%
***Acaryochloris* LARK001 (NIR)**	0.04634	0.605151	0.076576	92.9%

Table S1: Summary of Chl content in the *Acaryochloris* strains grown in NIR and white light.
